# *Description of a* Disease *Incident to the* Lymphatics, *and a New Species of Anatomical Injection*

**Published:** 1805-07-01

**Authors:** 

**Affiliations:** Anatomist, of New York


### *Description of a* DISEASE *incident to the* LYMPHATICS, *and a new Species of Anatomical Injection; by Mr.* RAMSAY, *Anatomist, of New York.*

PERMIT me, through the medium of your useful work,* to offer to the consideration of your learned readers a case, I believe, hitherto unnoticed, to which the lymphatic veins are liable. This discovery I made while a pupil of the justly-celebrated Cruikshank, of London. The year I forget, as my notes on this subject are in Europe.

Every anatomist must have observed, that lungs which have been subjected to violent and protracted morbid action, have often their lymphatic veins conspicuously dilated and opake. In forming a preparation of that organ, in this state, I was struck with the obstructions the quicksilver met with, more especially where the vessels, by their thickened coats, distension, and perfect whiteness, promised a happy injection. Upon minute attention to the cause, I was convinced that the sides of the vessels had grown together. For many years, especially after I became a teacher of anatomy at Edinburgh, where a large field of practical anatomy offered me opportunities of observation, this part of pathology became an object of my particular attention; and I seem to have detected, not only the incipient, but progressive and highly-diseased stages of the lymphatics in all parts of the system.

It is natural that the human mind, in such striking phenomena, which offer an ample scope to the most interesting consequences, should endeavour to scan the cause, and, if possible, prescribe the cure, or alleviate the evil. I shall only take the liberty, however, of here suggesting what has occurred to me, so far as the disease assumes different stages, and why the lymphatic veins may, by their peculiar offices, be liable to this peculiar affection.

The sanguiferous veins are liable to a thickening of the coats, so as frequently to assume the open mouth of an artery; and this often seems to occur in subjects where the

---

* The Medical Repository, 1804, from which this paper is extracted. Edit.

the arterial system is languid. I have supposed, in such cases, the appropriate arteries, which nourish the veins, are excited to an increased action, by the stimulus occasioned by congestion in the veins. In such veins I have distinctly demonstrated the muscular coat. There can be no accretion, however, of the cavity of the sanguiferous veins, as they continually receive their contents by continuity of canal, from their corresponding arteries. But there is a marked difference between the sanguiferous and lymphatic veins; the latter receiving their contents from the surfaces of the system, the propagation taking place by some means we seem not fully to comprehend, though it is possible, as the blood seems the appropriate stimulus of the arteries, the lymph becomes the appropriate stimulus of the lymphatic veins. That these vessels have muscles I believe, which are roused into action by their contents; and every day proves to us, that they, and their glands, possess the highest irritable principle, from the indurations of glands, and dilatation of the veins, where the lymph is tainted with any noxious quality.

If I am correct in the existence of this disease, it may explain many morbid phenomena. Justly celebrated authors have supposed a peculiar principle of life in the blood. If my memory serves me, some one draws inductions from the coagulum found in an aneurismal sac, which had been included by the ligatures of operation; and this coagulum was supposed to be found unchanged after years had elapsed. May not lateral branches of arteries and veins of such a sac have been conveying new blood continually, the lymphatics carrying off the superabundance, and the coagulum found be really more recent than is supposed, but coagulating constantly from the inaction of the sac? The vital principle of the blood appears to me not only plausible, but true; and I see not why the idea may not be extended to the lymph. In such lungs, however, as I injected, the lymph seemed in some peculiar morbid state; and when it is propagated towards a portion in a state of accretion, as this fluid is prevented from returning retrograde by the valves, if lateral branches are not sufficiently capacious to carry off the fluid, there must be a real stagnation; and several eminent practitioners have remarked to me, since mentioning the circumstance in my lectures at Edinburgh, that they had traced diseases, ulcers, &c. from seemingly this cause.

I have been used to offer, as a practical hint on the two venous systems, which are indirectly affected and dependent on the state of their corresponding lymphatic and sanguiferous guiferous arteries*, that we should aim at producing tone in the arterial system, by the stimulus of air, exercise, warm or cold bath, friction, &c. in preference to applications of violent medicines by the stomach, or mercurial frictions; by which means I have often succeeded in exhibiting medicines, when the system had acquired general vigour. As congestion in the sanguiferous and lymphatic veins destroy these valvular apparatus, their contents are not only not supported (which occasions effusions) but the fluids are not propagated so as to return and nourish the system. Exercise, then, or friction (avoiding the least approximation towards fatigue) in the course of circulation, where natural action is wanting, empties the veins, often banishes pain and anxiety, exhilarates the system, by propelling the contents, exciting action and nutrition, and checks dilatation of the vessels.

I shall now add an anatomical injection I have lately contrived, which I have not yet brought to that perfection I wish; but it is my duty, as a citizen of the world, to sacrifice my pride to the general benefit, relying on the indulgence of a generous community, whose extensive means may ripen our feeble attempts into real advantages. The very fine injections for anatomical purposes, composed of cinnabar and varnish, do not really always give a true idea, as they produce effusion, which may please the vulgar eye, without illustrating science. The wax injections, in some respects, are inconvenient, from the heat they require, as well as the warmth to which the subject is usually exposed, which often destroys the natural structure and appearances. The following injection seems to run minutely, and keep fluid a sufficient time to enable us to inject leisurely (very important in some cases which wax injections forbid);—it requires no heat, neither does the subject; and although thrown fluid and cold into the vessels, it hardens in thirty minutes or less. It is rather brittle. Chemistry may some day obviate this. It is not, however, so subject to expansion as the wax; hence it may be exposed to the greatest heats, in warm climates, without endangering extravasation or rupture. I have only tried this injection with the calces of lead, as the colouring ingredients;

* I divide the veins into sanguiferous and lymphatic; and the extreme rami of the arteries into effusori, or *open-mouthed arteries*, which include every species of nourishing, lubricating, or excreting arteries; and *sanguiferous* arteries, which, by continuity of canal, convey the blood to the veins.

dients; but, I presume, any calx or pigment may be found equally effectual. The calx is reduced to a very fine state and mixed with linseed oil, the same as found in the shops for painting. This, however, seems most successful when the paint is of a very thick consistence, admitting the more readily of the other ingredients to be mentioned. Mix the paint with a varnish composed of equal weights of spirit of turpentine and rosin, till it acquires the consistence of cream; saturate the fluid with water, of which it takes up a considerable quantity; throw it into the vessels, and it will harden in about half an hour, if the proportions are just. I suppose, as the hardening depends on the water, moistening the calx with water, previous to grinding it with the oil, will be a more simple process, and equally effectual as the above.

